# Prophylactic Cranial Irradiation May Impose a Detrimental Effect on Overall Survival of Patients with Nonsmall Cell Lung Cancer: A Systematic Review and Meta-Analysis

**DOI:** 10.1371/journal.pone.0103431

**Published:** 2014-07-29

**Authors:** Shuan-shuan Xie, Ming Li, Cai-cun Zhou, Xiao-lian Song, Chang-hui Wang

**Affiliations:** 1 Department of Respiratory Medicine, Shanghai Tenth People’s Hospital, Tongji University, Shanghai, Peoples R China; 2 Department of Oncology, Shanghai Pulmonary Hospital, Tongji University School of Medicine, Tongji University Cancer Institute, Shanghai, Peoples R China; Johns Hopkins Hospital, United States of America

## Abstract

**Purpose:**

To determine the role of brain metastases (BM) and overall survival (OS) in patients with non-small cell lung cancer (NSCLC) by performing a meta-analysis of the RCTs (randomized controlled clinical trials) and non-RCTs (non-randomized controlled clinical trials) published in the literature.

**Methods:**

A meta-analysis was performed using trials identified through PubMed, EMBASE and Cochrane databases. Two investigators independently assessed the quality of the trials and extracted data. The outcomes included BM, OS, median survival (MS), response rate (RR), Hazard ratios (HRs) and odds ratios (ORs), and their 95% confidence intervals (CIs) were pooled using ReMan software.

**Results:**

Twelve trials (6 RCTs and 6 non-RCTs) involving 1,718 NSCLC patients met the inclusion criteria. They were grouped on the basis of study design for separate Meta-analyses. The results showed that prophylactic cranial irradiation (PCI) reduced the risk of BM as compared with non-PCI in NSCLC patients (OR = 0.30, 95% [CI]: 0.21–0.43, p<0.00001). However, HRs for OS favored non-PCI (HR = 1.19, 95% [CI]: 1.06–1.33, p = 0.004), without evidence of heterogeneity between the studies.

**Conclusion:**

Our results suggest that although PCI decreased the risk of BM, it may impose a detrimental effect on OS of NSCLC patients.

## Introduction

Lung cancer is the leading cause of cancer-related mortality in both men and women. Approximately 221,130 new cases and 156,940 deaths are reported each year in the United States and about 1.3 million deaths worldwide. NSCLC accounts for about 85% of all lung cancers, and the 5-year survival of patients with metastatic NSCLC is less than 10% [1]–[3].

Brain metastasis (BM) occurs frequently in patients with NSCLC, especially in younger patients (<60 years) who underwent PCI and those with adenocarcinoma and large-cell carcinoma. The incidence of BM ranges from 17% to 54% as the first site of recurrence in 15–40% cases [4]–[6], and this risk is more than 50% in patients with small cell lung cancer (SCLC). Even though most patients could achieve some palliation after whole-brain irradiation, more than 50% of them could die from intracranial progression; and the mean survival (MS) is reported to be only about 3 to 6 months [7]–[11]. BM is therefore a common and devastating event in patients with NSCLC with a poor outcome. Since 1970s, PCI has been explored as a therapeutic option to lower BM.

Unlike SCLC, few randomized studies have addressed PCI in NSCLC. The first trial was conducted by the Veterans Administration Lung Study Group (VALG) [8], which showed that the incidence of BM was significantly lower in the PCI arm as compared with the observational arm (6% vs. 13%, *p* = 0.038), and that PCI had no effect on overall survival (OS). More surprisingly, another randomized trial conducted by the South West Oncology Group (SWOG) [12]–[13] included 254 patients with inoperable Stage III NSCLC; of whom, 226 were evaluable. The incidence of BM in the PCI arm was 1% *vs*. 11% in the observational arm (*p* = 0.003). However, OS was higher in the observational arm (8 *vs.* 11 months, *p* = 0.004). Even though most studies reported strong evidence in favor of PCI by virtue of reducing the incidence of BM by 50% in patients with NSCLC, its impact on OS remains uncertain and controversial with respect to indications of PCI in these patients.

Hence, the present systematic review and meta-analysis aimed to evaluate the potential role of OS and BM in PCI in patients with NSCLC as reported by RCTs and nonRCTs published in the literature.

## Materials and Methods

### Search Strategy

An electronic sensitive search of PubMed, EMBASE and CENTRAL (Cochrane Central Register of Controlled Trials) database were performed in August 2013, using the following key words as the search terms: “NSCLC”, “non-small cell lung cancer”, “non-small lung neoplasm”, “PCI”, “prophylactic cranial irradiation”, “prophylactic skull radiotherapy”, “brain irradiation (EBI)”, “overall survival”, “Brain metastasis”, “OS”, “BM”. Both RCTs and non-RCTs that fulfilled the criteria of a highly sensitive filter were included in this Meta-analysis [14]. The published languages and years were not limited. References of all randomized clinical trials were scanned for additional study. The American Society of Clinical Oncology (ASCO) and the European Society for Medical Oncology (ESMO) annual meeting abstracts in the latest 15 years were also searched.

### Selection Criteria

Trials were excluded if they did not meet with the following inclusion criteria. Trials were included if they 1) compared PCI with non-PCI; 2) enrolled NSCLC patients; and 3) reported results on OS and BM regardless of the publication status (published, conference proceedings, or unpublished).Two investigators (SX. and ML.) independently inspected each reference and applied the inclusion criteria. For possibly relevant articles or in case of disagreement, both investigators inspected the full text independently.

### Data Extraction And Quality Assessment

The two investigators independently extracted data from all primary studies that fulfilled the inclusion criteria, and any disagreement was resolved by consensus. In articles where outcomes were not reported, attempts were made to contact the authors for additional information. The following data were abstracted from each article with a standardized approach, including publication details, quality scores, trial characteristics (such as the first author’s last name, year of publication, number of lung cancer cases, primary therapy,stage, and the PCI dose), outcome measures (such as HRs for OS, OR for BM, and their 95% CIs, log-rank test, and *p* values).

The same reviewers independently assessed trials for methodological quality, and any disagreement was resolved by consensus. The methodological quality of each RCT was assessed using the Cochrane collaboration’s tool for assessing risk of bias [14], which utilizes seven aspects: I) details of the randomization method; ii) allocation concealment; iii) blinding of participants and personnel; iv) blinding of outcome assessment; v) incomplete outcome data; vi) selective outcome reporting; and vii) other sources of bias, to provide a qualification of risk of bias. Each of the seven items is scored as “low risk,” “unclear risk,” or “high risk”. Meanwhile, the included and case-control studies were assessed based on the 9-star Newcastle-Ottawa Scale for quality of non-randomized studies in meta-analyses.

### Statistical Analysis

Data were analyzed using Review Manager (RevMan, Version 5.0, Copenhagen: The Nordic Cochrane Centre, The Cochrane Collaboration, 2008). Time-to-event data were summarized by the log HR and its variance using previously reported methods [15]. Results were presented as HRs and 95% CIs using a general variance-based method. Dichotomous data were compared using an OR. Respective 95% CI was calculated for each estimate and presented in forest plots.

Statistical heterogeneity of the trial results was assessed with the *x^2^* test for heterogeneity and the *I^2^* test for inconsistency [14]. If the *p* value was less than 0.1 (*x^2^* tests), the results were considered heterogeneous; if the *I^2^* was greater than 50%, the results were considered inconsistent [16]. If the test results for heterogeneity were significant, the DerSimonian and Laird random-effects model was used to analyze the treatment groups [17]. The potential presence of publication bias was evaluated visually by inspecting funnel plots and statistically by the Egger’ test [*18*] *.*

## Results

### Search of the published literature

**Figure 1:**
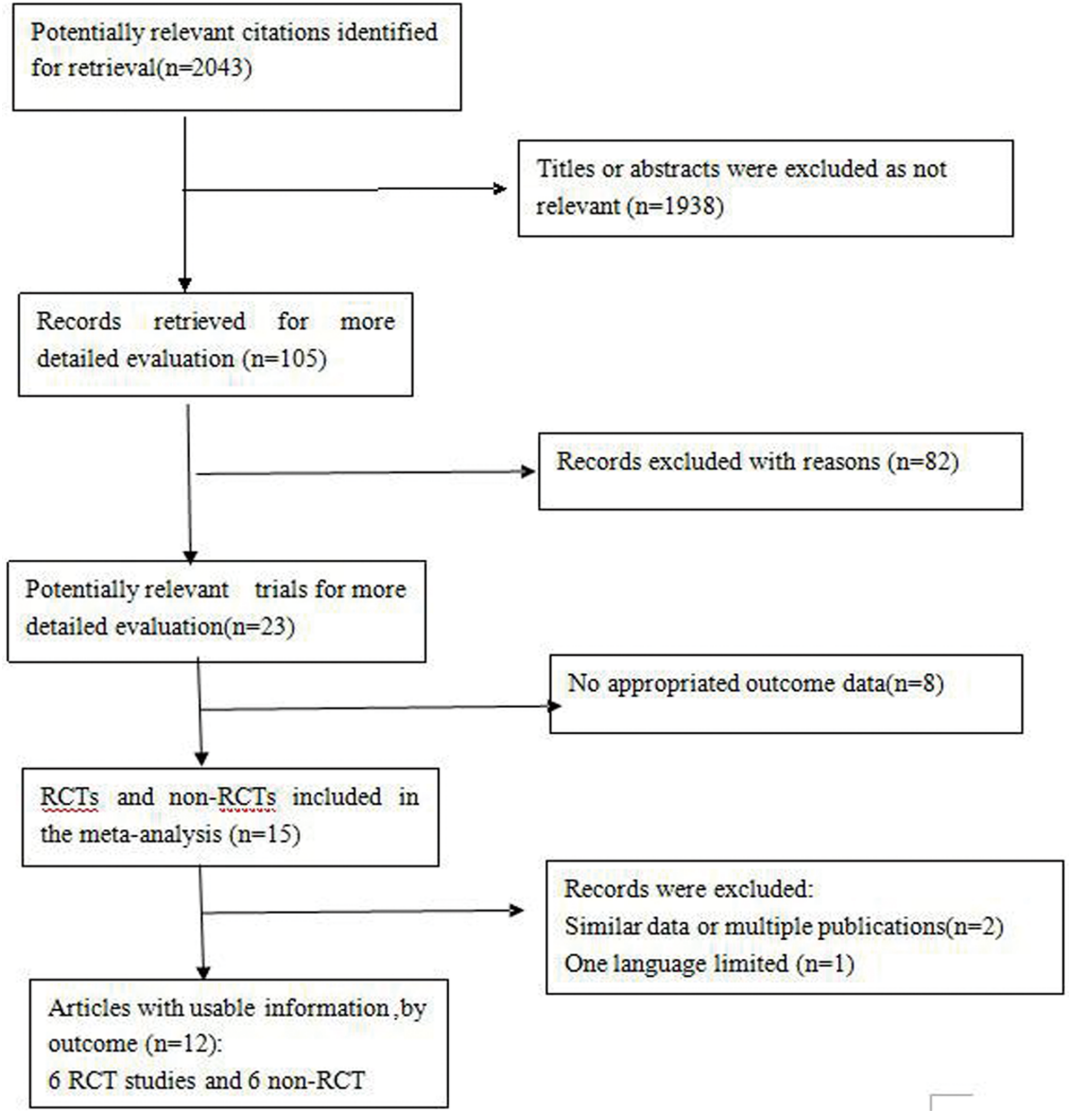
Procedures used for trial selection. Abbreviations: RCT,randomized controlled trial; Non-RCT, non-randomized controlled trial.

The systematic literature search identified 2,043 publications on PCI; of which, 15 trials included patients with NSCLC. After excluding 3 studies, 12 trials were included in this analysis, involving a total of 1,718 NSCLC patients [4], [5], [8], [12], [13], [19]–[27]. Figure 1 shows the reasons for exclusion of studies. None of the conference abstracts met the inclusion criteria, and therefore were not included for analysis. Secondary publications of previous reports were excluded, though any relevant and unique results were extracted and included.

Bias risk of each items for included RCTs are provided in Table 1. Most of the items were at “low risk” based on Cochrane handbook, but none of them specified the use of a double-blind methodology. Table 2 summarizes the quality scores of case-control studies based on the Newcastle-Ottawa Scale. Most of the observational studies scored 5 or more, suggesting a reasonably good quality of the case control studies.

**Table 1:** Methodological Quality of Included Randomized Controlled Trials*.

Study	Random sequencegeneration	Allocation Concealment	Blinding ofparticipant andpersonnel	Blinding ofoutcomeassessment	Incomplete outcome data addressed	Free of selective reporting	Free of other bias↑
Cox [8]	Unclear	Yes	Yes	No	No	Yes	Yes
Umsawasdi [19]	Unclear	Unclear	Yes	No	No	Unclear	Yes
Russell [20]	Yes	Yes	Unclear	No	Yes	Yes	Yes
Miller [12], [13]	Unclear	Unclear	Yes	No	Yes	Yes	Unclear
Pöttgen [21]	Yes	Yes	Yes	No	Yes	Yes	Yes
Gore [22], [23]	Yes	Yes	No	No	Yes	Yes	No

*Yes, low risk of bias; Unclear, unclear risk of bias; No, high risk of bias.

**Table 2:** Methodological quality of included case–control studies based on the Newcastle–Ottawa Scale.

Case-control studies	Selection				Comparability	Exposure			Total score
	Adequate definition of cases	Representativeness of cases	Selection of controls	Definition of controls	Control for importantfactor oradditional factor	Ascertainment of exposure	Same method ofascertainment forcases and controls	Non-response rate	
**Jacobs** [24]		*	*	*	*	*	*		6
**Skarin** [25]	*	*	*	*	*	*	*		7
**Strauss** [4]	*	*	*	*	**	*	*		8
***Albain*** [5]	*	*	*	*	**	*	*		8
**Stuschke** [26]		*	*	*	*		*		5
**Budach** [27]	*	*	*	*	**	*	*		8

Additionally, trials were assessed with respect to the inclusion criteria, details of PCI treatment, and description of statistical methods, and these details were clearly described in all 12 trials.

### Included studies

The baseline characteristics of the 12 trials are listed in Table 1 and Table 2. The studies included slightly different patient groups. Seven trials [4], [5], [19], [21], [23], [25], [26] used trimodality (chemotherapy, radiation, and surgery) as the primary treatment, and four studies [8], [12], [13], [20], [27] used chemotherapy or radiation therapy (except Jacob’s study) (Table1). The dose of cranial irradiation ranged from 30 to 37.5 Gy (except in Cox’s study where it was 20 Gy) (Table 1 and Table 2). Five RCTs required disease stage confirmation of the diagnosis (except Cox’s details of disease stage). All RCTs and nonRCTs trials reported MS or OS rates (Table1 and Table 2).

### RCTs on PCI in patients with NSCLC

Six RCTs [8], [12], [13], [19]–[23] evaluated PCI in NSCLC. In most trials, the cumulative incidence of BM was reduced in the PCI arm as compared to the control arm, but the impact on OS and MS remained unclear (Table 1).

PCI did significantly reduce the incidence of BM in five trials [8], [12], [13], [19], [21]–[23]. In the Cox [8] study, the incidence of BM was significantly lower in the PCI arm compared to the observational arm (6% *vs*. 13%, *p* = 0.038, Fisher’s exact test). In the Umsawasdi trial [19], the incidence of BM in the PCI arm was 4% compared to 27% in the observation arm (*p* = 0.02, chi-squared). In the Miller [12], [13] trial, the incidence of BM in the PCI arm was 1% compared to 11% in the observation arm (*p* = 0.003, chi-squared). Pöttgen [21] and Gore [22], [23] had also reported significant difference in the incidence of BM between PCI and observational groups (9.1% *vs*. 27.2% *p* = 0.04 and 7.7% *vs*. 18% *p* = 0.004). However, in Russell’s study [20], PCI did not significantly reduce the incidence of BM compared to the observational arm (9% *vs* 19%, *p* = 0.10, chi-squared).

The first trial of Cox et al [8] reported that MS was 8.2 months in the PCI group and 9.7 months in the observational group (*p* = 0.5, Gehan-Wilcox on test), while it was reported as 8.4 months and 8.1 months, respectively (*p* = 0.36, log rank test) by Russell et al. [20] Thus, there was no statistically significant difference between the two groups. However, Miller et al [12], [13] reported that MS was lower in the PCI arm than that in the observational group (8 months *vs*. 11 months, *p* = 0.004, log rank test).

Umsawasdi et al [19] reported that 3-year survival in the PCI and control groups was 22% and 23.5%, respectively; and there was no statistically significant difference between the two groups. Russell et al [20] also reported no significant difference in 1-and 2-year survival rates between the PCI and observational groups (40% *vs.* 44% and 13% *vs.* 21%, *p* = 0.36, log rank test). Pottage et al [21] conducted their trials in two local therapy options (Arms A and B), where in all patients in arm B received PCI. They found that there was a significant reduction in the probability of BM as the first site of failure (7.8% at 5 years *vs*. 34.7%, *p* = 0.02). In Gore’s [22], [23] trial, 3-year survival in the PCI and control groups were 26.1% and 24.6%, respectively (Table 3).

**Table 3:** Randomized Controlled Trials Evaluating PCI.

						Brain Metastases (%)			Median Survival (months)/Overall Survival (%)		
Study	Year	PrimaryTherapy	Stage	Dose (Gy)	N	PCI (+)	Observation	P	PCI (+)	Observation	*p*
Cox [8](VALG)	1981	RT only(all NSCLC)	Inoperable	20 (2 Gy×10)	281	7/136(6%)	16/145(13%)	0.038	8.2 months	9.7 months	0.5
Umsawasdi [19](MDACC)	1984	Trimodality(all NSCLC)	I–II (13%)III (87%)	30 (3 Gy×10)	97	2/46(4%)	14/51(27%)	0.002	22%(3 Years)	23.5%(3 Years)	NA
Russell [20](RTOG)	1991	RT only(nonsquam)	I/III	30 (3 Gy×10)	187	8/93(9%)	18/94(19%)	0.1	8.4 months 40%(1 Year)13% (2 Years)	8.1 months44% (1 Year)21% (2 Years)	0.36
Miller[12], [13](SWOG)	1998	Ctx+RT(all NSCLC)	III	30 (2 Gy×15)37.5(2.5 Gy×15)	226	1/111(1%)	13/115(11%)	0.003	8 months	11 months	0.004
Pöttgen[21](GMRT)	2007	Trimodality(all NSCLC)	IIIA	30 (2 Gy×15)	106	5/55(9.1%)	9/51(27.2%)	0.04	7.8%(5 Years)	34.7%(5 Years)	0.02
Gore[22], [23](RTOG)	2012	Trimodality(all NSCLC)	III	30 (Gy×15)	340	13/163(7.7%)	32/177(18%)	0.004	26.1%(3 Years)	24.6%(3 Years)	NA

### NonRCTs on PCI in patients with NSCLC

Six nonRCTs [4], [5], [24]–[27] had demonstrated the potential effect of PCI in patients with NSCLC (Table 4).

**Table 4:** Retrospective and Non-randomized Prospective Trials Evaluating PCI.

					Brain Metastasis						
					PCI (+)		Observation				
						Patients (n = )		Patients (n = )		N-year	Median Survival
Study	Year	Primary Therapy	Dose (Gy)	N	%	Total	%	Total	*P*	Overall Survival (%)	(months)
Jacobs [24]	1987	NA	30(2 Gy×15)	78	5	1/20	24	14/58	0.06	NA	17 months
Skarin [25]	1989	Trimodality(all NSCLC)	36(2 Gy×18)	34	14	1/7	26	7/27	NA	31 % (3–5 Years)	32 months
Strauss [4] ^ (CALGB)^	1992	TrimodalityNon-epidermoid	30(2 Gy×15)	54	0	0/13	12	5/41	0.32	58 %(1 Years)	15.5 months
Albain [5] (SWOG)	1995	Trimodality(all NSCLC)	36(2 Gy×18)	126	8	2/26	16	16/100	0.36	37% (2 Years)27% (3 Years)	15 months
Stuschke[26]	1999	Trimodality(all NSCLC)	30(2 Gy×15)	75	13	6/47	54	15/28	0.0004	31% (3 Years)	20 months
Budach [27]	2003	Ctx/RT	30(2 Gy×15)	104	2	1/54	20	12/60	NA	NA	NA

Abbreviations: PCI, prophylactic cranial irradiation; TX = treatment; RT, radiation therapy; Ctx, chemotherapy; Trimodality, chemotherapy+radiation therapy+surgery; NA, not available; NSCLC, non–small-cell lung cancer; SWOG, South West Oncology Group; CALGB, Cancer and Leukemia Group.

The first trial was conducted by Jacobs et al, [24] who reported that the BM in PCI and control groups was 5% and 24%, respectively; and there was no statistically significant difference between the two groups (*p* = 0.06). Strauss et al [4] and Albain et al [5] also reported no significant difference in BM between the two groups (0% vs. 12%, P = 0.32 and 8% vs. 16% *p* = 0.36). Skain [25] and coworkers treated 34 patients with stage III NSCLC with chemotherapy and radiation followed by surgery. About 14% of patients received treatment with PCI developed BM compared with 26% of patients who did not receive PCI treatment.

In the most notable study by Stuschke trial [26], 75 patients with stage IIIA/IIIB NSCLC received treatment with induction trimodality (chemotherapy, radiation therapy, and surgery). PCI was introduced after the first half of the study because of a high incidence of brain relapses. Patients treated during the second half of the study were offered PCI (30 Gy in 15 fractions). In an otherwise uniformly-treated, uniformly-staged cohort of patients, they found that there was a significant reduction in the probability of BM from 54% to 13% (*p*<0.0001).

### Analysis on BM

**Figure 2:**
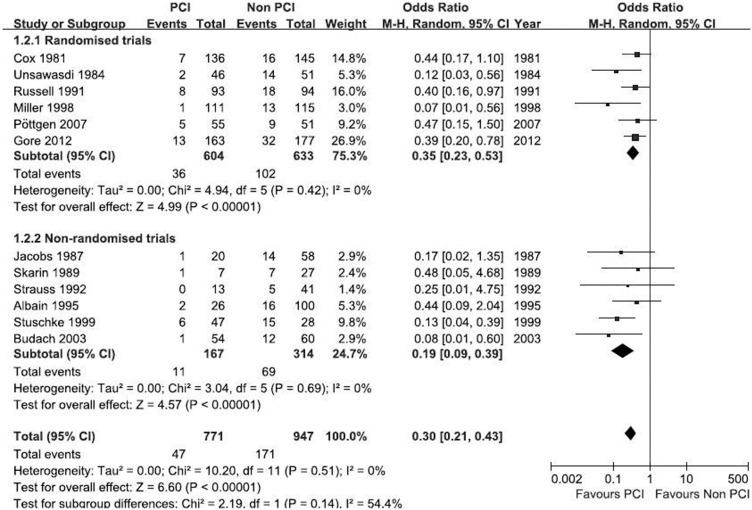
Results of the meta-analysis on studies evaluating the effect of PCI on brain metastases: OR: 0.30 (95% CI: 0.21–0.43).

**Figure 3:**
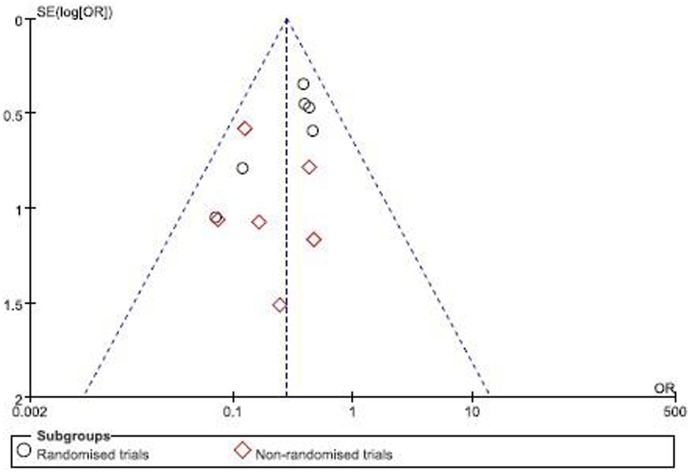
Results of the meta-analysis on studies evaluating the effect of PCI on overall survival: HR: 1.19 (95% CI: 1.06–1.33).

The meta-analysis on BM included 12 trials involving a total 1,718 patients including 771 patients who received PCI. All the 12 trials reported the impact of PCI on BM, concluding that PCI was associated with a significant reduction in the overall mortality of patients with NSCLC as compared with those who did not receive PCI (OR = 0.30; 95% CI: 0.21–0.43; *p*<0.00001). There was no heterogeneity across studies (I^2^ = 0%; *p* = 0.51) (Figure 2), indicating that the results were valid. In addition, no publication bias was detected by Egger’s test. The funnel plot is shown in Figure 3. There were no asymmetry observed, suggesting absence of any publication bias.

### Overall survival (OS)

**Figure 4:**
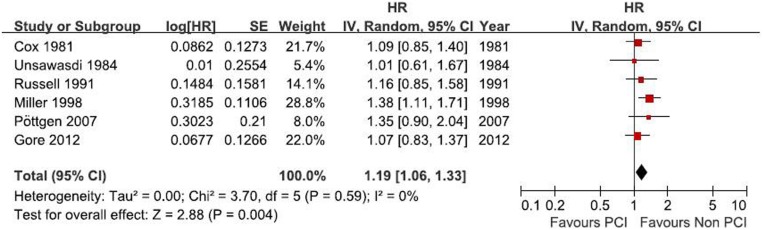
Results of the funnel plot of the studies evaluating the effect of PCI on brain metastases: OR, Odds ratio.

**Figure 5:**
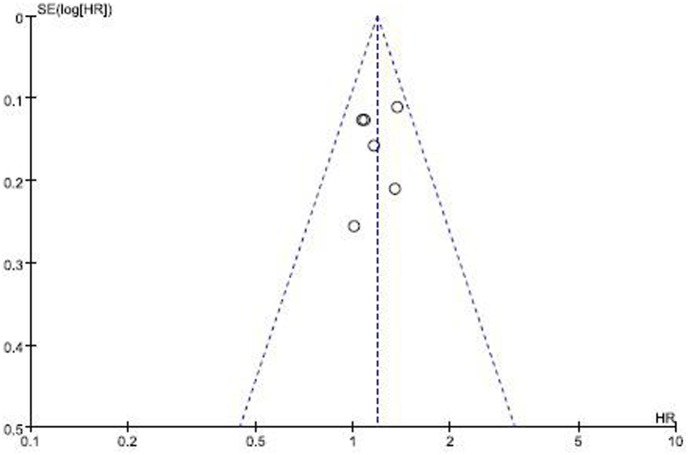
Results of the funnel plot of studies evaluating the effect of PCI on overall survival: HR, Hazard ratio.

All the six RCTs (comprising 1,237 cases) reported hazard ratios (HRs) for OS. The HR for OS favored treatment without PCI (HR = 1.19, 95% CI: 1.06–1.33 *p* = 0.004), without any evidence of heterogeneity between the studies (I^2^ = 0%; *p* = 0.59) (Figure 4). The pooled HR for OS was performed using the fixed-effort model. The result indicated that PCI unfavorably affected OS (risk of death: 19%) as compared with patients who did not receive PCI. In addition, no publication bias was detected by Egger’s test.The funnel plot is shown in Figure 5. There was no publication bias since the studies show symmetry.

## Discussion

This meta-analysis provided additional insights into use of PCI in patients with NSCLC. The analysis included 12 clinical studies (6 RCTs and 6 nonRCTs), involving a total of 1,718 patients with NSCLC. All trials compared treatment of NSCLC with and without PCI. As shown by the meta-analysis, PCI reduced the risk of brain metastases as compared with patients who did not receive PCI (OR = 0.30, *p*<0.00001). However, the HRs for OS favored non-PCI modality (HR = 1.19, *p* = 0.004). In addition, the data currently available are not sufficient and convincing enough to make a definitive conclusion about the effect of PCI on toxicity and radiation dose in patients with NSCLC. Thus, it remains unclear whether PCI could cause toxicity and result in a decline in neurocognitive function (NCF) or quality of life (QOL).

### Other studies

A previous systematic review published in 2010 identified four published RCTs [8], [12], [13], [19], [20], it was unable to draw a warranted statistical conclusion. Results showed that there was a significant reduction in the cumulative incidence of BM varying from 50% to 90%. However, data were not sufficient enough to perform a meta-analysis due to small sample size, and therefore only a narrative synthesis was performed without reporting any benefit concerning OS of patients who received PCI. Recently, a large study including 2,360 patients with lung cancer [28] reported that there was a significant decrement in OS associated with PCI, with a 2-year OS of 14% *vs*. 28% and a 5-year OS 5% *vs.* 12% in PCI *vs*. non-PCI groups (*p*<0.01). These findings are consistent with the results of present work.

In most randomized trials, the cumulative incidence of BM was reduced in the PCI arm as compared with the control arm, but the impact on OS remains undetermined. In a randomized trial reported by Gore et al [22], [23], patients were randomized between PCI (30 Gy in 15 fractions) or non-PCI groups. However, the trial was closed due to poor accrual (358/1058 patients needed). The results showed a significant reduction in the incidence of BM from 18 to 7.7% in the PCI group at 1 year (HR 0.43 in favor of PCI, 95% CI 0.23–0.78, *p* = 0.004) and nonsignificant trend towards an increased relapse-free survival at 1 year (51.2 and 56.4% for observation and PCI, respectively, *p* = 0.11). There was no significant difference in OS between the two groups (hazard ratio 0.97, 95% CI 0.74–1.30, *p* = 0.86). As a result, these findings failed to confirm the role of OS in PCI in patients with NSCLC. Therefore, the present meta-analysis included more RCTs and nonRCTs and attempted to include all currently available data so as to assess the potential effect of PCI-related OS and BM in patients with NSCLC. The results suggest that OS is worse in patients of the higher-dose PCI group, or PCI may impose potential neurological hazard.

### PCI dose

In SCLC, few trials [36], [37] suggest that PCI should be administered at the dose of 25 Gy in 10 fractions when the patients have good response to first-line treatment In limited disease SCLC. In extensive disease SCLC, the recommended PCI dose to patients who responds to first-line chemotherapy is 20 Gy in 5 weeks. However, it is unable to establish the most effective regimen of radiotherapy till date for PCI dose in NSCLC due to the availability of few trials to consider.

Three trials that showed a significant reduction in the incidence of BM with PCI used different regimens. The Umsawasdi et al [19] trial used 30 Gy in 10 fractions over two weeks and the Cox et al [8] trial 20 Gy in ten fractions over two weeks. The Miller et al [12], [13] trial used 37.5 Gy in 15 fractions for the first 34 patients and 30 Gy in 15 fractions for the remaining 77 patients; there was no significant difference in MS between the two PCI regimens used. The differences in inclusion criteria made any comparison between the trials inappropriate. In addition, no randomized trial had compared these (or any other) PCI regimens head-to-head; hence, it was not possible to conclude which was more effective.

### Toxicity and QOL

The meta-analysis showed that PCI prevented or delayed the incidence of BM, but it did harm the OS of patients with NSCLC. In addition, few trials reported that PCI could cause toxicity resulting in a decline in NCF or QOL in Table 5. Therefore, the indications of PCI should be considered in the light of its potential neurotoxicity. Unfortunately, long-term neurotoxicity was not adequately described in the present analysis. However, several studies have reported neurological and intellectual impairment or abnormalities on brain computed tomography scan, which are potentially related to PCI that can be of concern to clinicians. For instance, acute toxicity mostly includes alopecia, headache, fatigue, nausea, and vomiting. Long-term sequelae such as severe memory loss, intellectual impairment, or even dementia and ataxia have been reported in retrospective studies and attributed to PCI (Table 5).

**Table 5:** Prospective Studies With Toxicity and QOL Evaluating PCI in NSCLC.

Study	n	PCI dose(Gy/fraction)	Patientsreceiving PCI	Neuropsychological tests	Baseline evaluation Impairment (%)			Evaluation after PCI	Impairment after PCI
					PCI	Observation	P		
Umsawasdi[19]	97	30/10	randomization	clinical findingS, CT	Not stated			Evaluated but not stated	NS but only 1 patient developed transient memory loss for 2.5 weeks.
Russell [20]	187	30/10	randomization	neurological physicalexaminations, CT	Not stated			At intervals of 3 months.	NS other than epialtion and skin reactions.
Pöttgen [21]	11	30/15	randomization	Trail-making A and B, Wechsler memoryscale, Benton visual retention,achievement measure, Corsi block tapping,Attention test battery, MRI	Not stated			Once after at least 4 years	NS but very small number of patients
Sun [29]	340	30/15	randomization	NCF:	NA:	NA:	NA:	At 3, 6, and 12 months	NS in MMSE or QOL after PCI, but there was a significant decline in HVLT at 1 year.
				MMSE:	95%:	95%:	1:		
				HVLT:	93%:	95%:	0.44:		
				ADLS:	96%:	94%:	0.40:		
				QOL: EORTC- QLQC30/QLQBN20	NA	NA	NA		
					90%	92%	0.52		

PCI, prophylactic cranial irradiation; NS, not significant (in terms of neuropsychological impairment between PCI and observation patients); CT, computed tomography; NCF, neurocognitive function; MMSE Mini-Mental Status Examination; HVLT, Hopkins Verbal Learning Test; ADLS, Activities of Daily Living Scale; QoL, quality of life;EORTC, European Organization for the Research and Treatment of Cancer.

Most studies concerning the effects of PCI on NCF and QOL were conducted in patients with SCLC; and relevant data in patients with NSCLC are limited, mainly due to the lack of intensive NCF and QOL testing in NSCLC trials. The only study available on NCF and QOL was done by Sun et al [29], who reported that there was no significant difference in global cognitive function or QOL after PCI, but there was a significant decline in memory at 1 year as defined by the Hopkins Verbal Learning Test. Russell et al [20] reported no acute toxicity other than epialtion and skin reactions. Pöttgen et al [21] published a neurocognitive evaluation on 11 out of 17 long-term survivors of stage IIIA NSCLC treated with or without PCI; the study pointed out that neurocognitive late effects were not significantly different between patients treated with or without PCI, but their sample size was very small. Umsawasdi et al [19] reported no late complications of PCI. Miller et al [12], [13] reported no excessive neurological toxicity with PCI as compared with the observational arm, but the definition of neurological toxicity was not stated. Other RCTs and nonRCTs trials did not report any PCI-related toxicity.

To conclude, there are very limited data available regarding the effects of PCI on toxicity and QOL in patients with NSCLC. Furthermore, evaluating PCI neurological toxicity is difficult because the related symptoms can be caused by many different factors. Treatment modalities such as PCI dose and fractionation scheme (fraction size Gy) or use of concurrent chemotherapy may contribute to neurotoxicity [30]–[34]. Thus, no meta-analysis has been attempted due to insufficient information, and only a narrative synthesis was performed. Thus, clinicians need to make a choice by weighing the benefits and risks of the treatment to individual patients. More researches on the effects of PCI on neurocognitive function could promote the clinical value of PCI.

### Strengths and weaknesses of this review

To the best of our knowledge, this is the first and largest meta-analysis performed so far to evaluate the role of BM and OS in patients with NSCLC. This analysis has some advantages. First, it takes into account the difference in the design of the PCI and non-PCI therapy for patients with NSCLC. In addition, it combines data from a number of RCTs and nonRCTs that enrolled substantial patients, thus significantly increasing the statistical reliability.

There were several limitations in the study. Firstly, as only few data of patients with NSCLC were available for analysis and other data such as HRs for OS based on histology, gender, and age were not mentioned in most studies, further analysis of individual patient data is needed to confirm the study findings. Secondly, although publication bias was not found according to funnel plots and Egger’s test, the small number of trials and possible existence of unpublished studies limited the power of these tests. Furthermore, the method used to calculate HRs and different covariates used for adjustment of HRs may lead to potential bias. HR, log HR, and its variance were calculated from the data or survival curves included in the article. In addition, HRs in the studies were adjusted for different covariates, and covariates were not consistent even in multivariate analysis performed in different studies.

### Key takeaways from this review

BM impairs QOL and is associated with a poor prognosis [35]. The rationale behind PCI is to control or eradicate undetectable micrometastases before they become clinically significant without inducing severe adverse effects. The present work attempted to establish whether PCI could prevent the development of BM and increase survival in patients with NSCLC who received treatment with curative intent.

In conclusion, PCI has demonstrated to reduce or delay the incidence of central nervous system failure in patients with NSCLC; but it does harm patients’ OS, and it may cause neurotoxicity. However, this should be considered as an aggregated effect mainly driven by data from trials with inadequate concealment. While patients with SCLC derive benefits of both OS and BM from PCI [36]–[38], there is a considerably higher likelihood for intracranial metastatic disease in SCLC than in NSCLC. The present results imply that there may be a propensity threshold for intracranial metastatic disease below which PCI is not warranted. The future clinical trials investigating the role of PCI for NSCLC may not be justified [28]. Therefore, clinicians need to make a choice by weighing the benefits and risks to individual patients. If clinicians can select out patients who are fit to PCI, unwanted harmful effects on patients can be avoided and wastage of medical resources could be reduced.

### Unanswered questions

The results from this review cannot be considered conclusive, owing to the heterogeneous nature in patient’s selection, primary treatment, and PCI dose. In addition, the overall quality of the six randomized trials is not high enough, and they are small in size. Therefore, large scale clinical researches are to be conduced to study the potential effects of BM and OS in patients with NSCLC. The quantitative findings from this review may allow for a better planning of those trials, selection of trial end points, and sample size estimation.

## Conclusion

Although this review was limited to a small number of randomized controlled trials and retrospective cohort studies, it provides some evidence suggesting that PCI could decrease the risk of BM in patients with NSCLC to some extent. However, results also suggest that PCI may have a detrimental effect on OS. To further confirm the use of PCI universally, some high-quality and adequately-powered RCTs that focus on OS, toxicity, and PCI dose are urgently needed. The results of ongoing randomized trials may change this recommendation in the future.

## Supporting Information

Checklist S1 **PRISMA checklist.**
(PDF)
